# Conditions Associated with Childhood Asthma in North Texas

**DOI:** 10.5402/2012/823608

**Published:** 2012-09-24

**Authors:** Patricia Newcomb, Alaina Cyr

**Affiliations:** College of Nursing, University of Texas at Arlington, 411 South Nedderman Drive, Arlington, TX 76019, USA

## Abstract

*Introduction*. The purpose of this study was to identify significant associations between asthma diagnosis, comorbid conditions, and social problems in children. *Method*. This study explored data collected in a unique, regional survey of children's health in north Texas originally administered in 2009 to a random sample of 21,530 households with children from 0 to 14 years of age. Descriptive statistics were compiled for the subsample of children with asthma, associations of interest were identified, and strengths of relevant associations were calculated. *Results*. The prevalence of asthma in school-aged children in the target area is 19–25%, which exceeds both national and state values. Statistically significant associations were found between asthma and allergies, sleep problems, and tonsillectomy. Significant associations were identified between asthma and school absences, academic problems, and behavior problems in school. There was a significantly greater prevalence of obesity/overweight among children with asthma than without asthma. *Discussion*. Children with asthma are at high risk for impairment in multiple dimensions. Thorough assessment, including comprehensive medical, social, and environmental histories, is critical in management of pediatric asthma.

## 1. Introduction 

Asthma affects individuals of all ages, but in childhood, asthma is the most common chronic disease after dental caries [1]. It is an important public health problem in the United States and its impact is influenced by both genetics and environment. Geography is strongly associated with variations in asthma prevalence. Some densely populated areas in the United States, such as the District of Columbia, Maryland, and Hawaii, experience childhood asthma prevalence rates as high as 17–22% while less populated areas, such as Iowa, Montana, and Nevada, report childhood asthma prevalence as low as 8-9% [2]. There is little information on variations in regions smaller than states. As more investigations of complex interactions between genes, environment, and lifestyle are conducted, urban/regional development (the expansion of built environments into natural areas) as well as natural characteristics of regions may play a greater role in judging the relevance of research findings about children with asthma [3].

 Tarrant and six surrounding counties comprise the western end of the Dallas-Fort Worth urban corridor in Texas. This region is interesting in the study of childhood asthma because of high asthma prevalence, a history of poor air quality, rapid development, and abundance of natural aeroallergens relative to other regions. Furthermore, Tarrant and surrounding counties are situated in the Barnett Shale natural gas field, and Tarrant county leads Texas in natural gas production. Over 3000 wells have been drilled in Tarrant county, largely in urban areas, including residential neighborhoods [4]. Citizen concern regarding drilling prompted the city of Fort Worth in Tarrant county to commission an air quality study, which was published July 2011. The study reported no evidence of 24-hour concentrations of toxic chemicals reaching levels of health concerns as currently defined, but anxiety regarding long-term health consequences persists [5].

The Tarrant county Public Health Department, reporting 2009/2010 Behavioral Risk Factor Surveillance System (BRFSS) data, noted that just over 7% of Tarrant County children younger than 18 years had asthma currently [6]. Additional data was generated in 2009 by the Cook Children's Health System, which undertook the Community-wide Children's Health Assessment and Planning Survey (CCHAPS). CCHAPS data indicated that 18% of children under the age of 15 years in Tarrant county, and 17% of children in surrounding counties had ever had physician-diagnosed asthma. It reported a prevalence of ever having asthma up to 25% in some age groups, instantly capturing the attention of community health advocates and environmental activists [7].

**Purpose**

The purpose of this study was to describe associations between physician diagnosed asthma and health indicators/conditions in a random sample of children in a heavily developed area of Texas using data obtained from the 2009 CCHAPS survey.

## 2. Methods

This study was approved by the Cook Children's Health Care System Institutional Review Board. Data was obtained from the CCHAPS survey which was conducted by research contractors, ETC and Texas A&M University. The survey employed a random sample of 21,530 households with a child from 0 to 14 years of age yielding 7,349 responses, a 34% response rate. Respondents were caregivers, including parents, foster parents, or kinship caregivers, who identified one child from the household as the subject. Households were drawn from Tarrant and five surrounding counties in the north Texas region. The survey was stratified geographically to insure sufficient numbers of respondents from each county for statistical validity [7]. 

The survey was conducted in 2009 by means of telephone interviews, mailed surveys, and internet surveys. To reduce respondent burden, two versions of the survey were administered. Each version contained a set of identical core questions. Another set of questions about specific health problems of childhood were divided between the two versions resulting in two relatively short surveys rather than a single long survey. Selected results of the survey and a description of survey methods are posted on the CCHAPS website [7]. Results for core questions included in both versions of the survey are reported by ETC to be accurate within 1.1% (95% confidence level), while results for the version-specific questions are accurate to within 1.6% (95% confidence level). Details of questions examined for this analysis are shown in Table 1.

Frequencies were obtained for all variables and bivariate associations were quantified using chi-Square or Fisher exact tests for nominal variables. Cross-products of 2 × 2 tables were used to assess odds ratios and *t*-test was used to compare means of continuous variables, such as days of hospitalization, for children with asthma and those without asthma. Linear regression was used to model predictors for continuous outcomes and logistic regression for categorical outcomes. 

## 3. Results

### 3.1. Geographical Distribution of Asthma Cases

Children with asthma are distributed widely across the target area. Although there are fewer cases in rural areas, consistent with fewer residents, children with asthma were no more likely to live in the densest “inner city” urban cores than in more suburban areas. Some of the densest clusters of asthma cases occurred in middle-class and more affluent suburban settlements in highly developed Tarrant county. 

### 3.2. Prevalence

**Figure 1:**
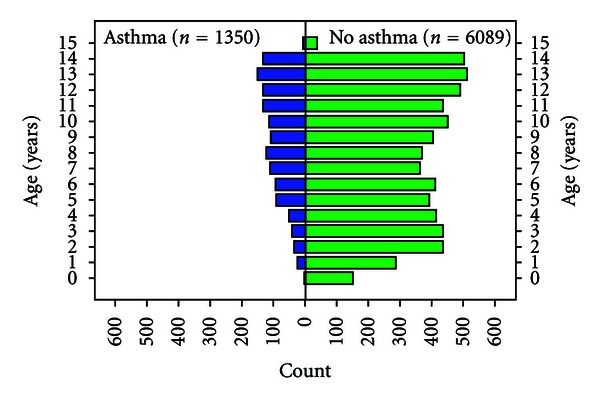
Distribution of children with and without asthma.

Children with the highest prevalence of ever having asthma, determined by caregiver report of physician diagnosis, were school-age children and adolescents. Prevalences ranged from 19% for 6 year olds to a high of 25% for 9 year olds. Prevalences for adolescents from 12 to 15 years of age ranged from 23.5% to 20.6%. Distribution of asthma cases by age is shown in Figure 1. 

For comparison, the national prevalence for diagnosis of childhood asthma as reported in the 2007 National Survey of Children's Health was 9% and Texas prevalence was 7% [8]. Earlier national data placed child asthma prevalence as high as 12% with peaks up to 15% for more heavily urbanized states [9]. Data from the American Lung Association [10] actually show prevalence of asthma in Texas children decreasing from 9.4% in 2006 to 8.2% in 2009.

### 3.3. Sample Characteristics

Highlights of the sample characteristics are included in Table 2. Of the children identified as having asthma, males outnumbered females almost 2 to 1. African-American/Black children constituted 6% of the entire sample, but composed 9% of the asthma subgroup. 

The association between self-identification as African-American/Black and having asthma was significant (odds ratio of 1.7, C.I. = 1.35–2.1, and *P* ≤ .0001). The proportion of Caucasians in the asthma subgroup was the same as the proportion of Caucasians in the entire sample. 

Of the parents reporting high school diploma or less as educational level, 15% had children with physician diagnosed asthma. In the group of parents with education past the high school level, 19% had children with asthma. Although educational level was significantly associated with child asthma diagnosis (*P* < .006), household income was not. Receipt of free school meals, a proxy for low-income status, was not significantly associated with asthma diagnosis either. Exposure to tobacco smoke was low for all children. Of the caregivers who were asked (*n* = 3768), 85% claimed children were seldom or never exposed to cigarette smoking at home. This estimate is consistent with CDC findings that 81% of Texas homes have “no smoking” rules [11].

### 3.4. Health Problems Coexisting with Asthma 

Children with asthma were no more likely than others to be perceived as having poor health; however, significantly fewer children with asthma were described as having excellent health than other children. Only 32% of children with asthma were described as having excellent health, while 58% of children without asthma were described as having excellent health (*P* < .0001). Children with asthma were found to be significantly more likely than other children to visit the emergency room (ER) frequently. They were 1.7-fold more likely to visit the emergency room 2 or more times in the past year and 2.4-fold more likely to visit 3 or more times in the past year (C.I = 1.5–3.6, *P* < .0001).

Although children with asthma visited the ER more frequently than others, they did not spend more days as inpatients. For children who were hospitalized once, only 8% were hospitalized for asthma. Among those who were hospitalized twice in the past year, only 7% were hospitalized the second time for asthma. Multivariable linear regression was used to model predictors for ER visits and showed that although the diagnosis of asthma predicted number of ER visits (*β* = 0.219; *t* = 6.999; *P* < .0001), other predictors were important, including parental perception of child's health (*β* = −0.093; *t* = −6.425; *P* < .0001), low economic status reflected in family receipt of food stamps (*β* = 0.107; *t* = 2.927; *P* = .003), and days spent in the hospital in the past year (*β* = 0.049; *t* = 11.869; *P* < .0001).

Infants, toddlers, and preschoolers may be hospitalized for wheezy lower respiratory problems, but older children are more likely to be admitted for asthma specifically, therefore we looked at hospitalizations for school-age/adolescent children separately. After removing 2 outliers who spent greater than 100 days in the hospital (not asthma), among children aged 5 years and older who were admitted to hospital (*n* = 258), mean days hospitalized were significantly less for children with asthma than for other children (*P* = 0.02). Furthermore, children with asthma were more likely to have medical insurance coverage than other children (OR = 1.5; CI 1.1–1.8; *P* < .02). Children with a diagnosis of asthma who were admitted to the hospital in the past year spent a mean of 4 days in the hospital, while children without asthma who were admitted to the hospital spent a mean of 6 days in the hospital. 

Given the allergic basis of much of childhood asthma, it was not surprising to find a strong association between physician diagnosed asthma and physician diagnosed hay fever/allergies in subjects for whom responses were available on both variables (*n* = 3672, *χ* ^2^ = 442.1, *P* ≤ .0001). Seventy-nine percent of children with asthma reported having hay fever or allergies. Children with asthma were 7 times more likely to have a diagnosis of hay fever/allergies than other children (OR = 7.2, CI = 5.9–8.8, *P* < .0001) as shown in Table 3.

Other significantly associated problems included sleep problems, tonsillectomy, and obesity/overweight. Both versions of the survey asked if the target child had a diagnosis of asthma made by a physician. One version of the survey included queries regarding sleep problems, tonsillectomies, and concerns regarding overweight for the target child. Responses to the latter survey of 3678 respondent parents/guardians indicated a 2-fold greater risk for having sleep problems for children with asthma than for those without asthma. Children with asthma or allergies were significantly more likely to receive tonsillectomy than children without asthma (*P* < .0001). Removing children with allergies from the analysis resulted in the same finding, that is, children with asthma who did not report allergies were significantly more likely to receive tonsillectomy than children without asthma (*P* < .0001). Children with asthma were about 3 times more likely than children without asthma to have their tonsils removed (OR = 2.9; CI 2.3–3.8) regardless of allergic status. 

In the total sample (*n* = 7439 respondents), 29% of children were classified by BMI as overweight or obese. For the subsample with complete responses for both asthma status and BMI (*n* = 5864), there was a significant association between BMI classification as overweight/obese and having a diagnosis of asthma (*χ* ^2^ = 6.2, *P* = .01). When the sample was stratified to examine school-aged children separately, the trend toward overweight/obesity in children with asthma was more pronounced. Among children 5 years of age or older (*n* = 4719), asthma and overweight/obesity were significantly associated (*χ* ^2^ = 15.2, *P* = .0001). The frequencies of overweight/obesity revealed that the percentage of children who were overweight/obese remained stable into school age for those with asthma, but dropped for children without asthma. There was also a significant difference in parental concerns that the target child was overweight: 15% of parents of children with asthma were concerned that the child might be overweight, while 10% of parents of children without asthma were concerned that the child might be overweight (*P* < .001). Parents/guardians of children with asthma were also less likely to agree that there are sufficient fitness opportunities for their children (*P* < .0001). Furthermore, within the group of children with asthma, there was a positive relationship between being overweight/obese and the number of hours children usually slept (*P* = .03). There was no relationship between hours of sleep and overweight/obesity among children without asthma. 

### 3.5. Social Problems of Children with Asthma

Among the sample of 3,592 school-aged children for whom the question was answered, asthma and allergies were independent and significant predictors of missed school days (*P* < .0001 for asthma and *P* < .0001 for allergies). Children with asthma were significantly (*P* = .04) overrepresented among children having academic problems in school; they composed 24% of children identified as having academic problems. In addition, children with physician diagnosed asthma were significantly more likely than children without asthma to have behavior problems in school (Fisher's exact *P* = .01).

## 4. Discussion

 For the purposes of this survey, asthma was defined as a diagnosis of asthma *ever* made by a physician or healthcare professional. The substantial difference in prevalence findings from the Tarrant county BRFSS and the CCHAPS is likely due to different outcomes being measured (*ever* had asthma versus *currently* have asthma). Some differences in sampling methods, such as mailing in addition to phone contact in the CCHAPS survey, and possibly the use of Mexican Spanish in Spanish language surveys used by the County and “traditional” Spanish used in CCHAPS surveys may have also played a role. 

Because childhood asthma is a disease characterized by highly variable symptom episodes, assessing current asthma status provides more information about asthma control than prevalence of the disease. Currently the only feasible goal of asthma treatment is to control asthma symptoms, not to cure asthma. From this perspective, the absence of current symptoms does not indicate that a child with a previous diagnosis of asthma is free of asthma. In fact, one of the major problems with adherence to treatment is family perception that absence of symptoms means the child has “outgrown” asthma. 

According to Environmental Protection Agency summaries of NCHS and NHIS data [12], the prevalence of lifetime diagnosis of asthma in children from birth through 17 years of age is 138 per 1,000 (14%) nationally. CCHAPS data indicates that prevalence of ever having asthma in Tarrant County and surrounding areas substantially exceeds national prevalences at 185 per 1,000 (19%) in Tarrant county and 174 per 1,000 (17%) in surrounding counties. Why prevalences in this region should be so high relative to other regions in the United States cannot be determined from cross-sectional data, but one possible influence driving childhood asthma prevalence is outdoor air pollution. 

Tarrant and three surrounding counties have been designated as being in nonattainment status under the 8-hour National Ambient Air Quality Standards (NAAQS) for ozone, and Tarrant county has been in nonattainment for over a decade. Unlike some other urban areas in Texas, such as the Houston area, air pollution in Tarrant and surrounding counties is largely the result of on-road and off-road (jets and construction vehicles) traffic, rather than fixed sources, like factories. Traffic emissions include high levels of particulates known to be capable of inducing airway inflammation and proximity to traffic increases risk for asthma [13, 14]. 

 Some evidence for the role of outdoor pollution in driving asthma prevalence was found in previously published findings by analysts in Cook Children's Health System showing that the bulk of Tarrant County asthma cases lie directly in the path of southeasterly winds that have historically carried high levels of particulate matter from working cement kilns in a neighboring county. Asthma prevalence increases in a linear configuration within the path of this “cement plume” as residential location comes closer to the cement kiln area [7]. Urban drilling for natural gas may play a role as well. Advances in horizontal drilling and hydraulic fracturing technologies have pushed natural gas production in north Texas cities to the highest levels in decades. Some components in the hydraulic fracturing process, such as methanol, benzene, toluene, xylene, and ethylbenzene, and others are known to be hazardous air pollutants. 

Indoor air pollution could be an influence as well, but it is unlikely that indoor pollution in north Texas differs in great degree from many similar areas of the USA with lower asthma rates. In fact, the geographic distribution of asthma cases in this survey is notable for the lack of clustering in “inner city” areas of concentrated poverty, which are thought to have worse indoor air pollution than more prosperous areas due to deteriorating housing stock. On the other hand, failure to adequately sample the lowest income strata may have contributed to lack of clustering in lower income areas, as well.

Finally, the role geography plays in asthma prevalence may be influenced by regional variations in healthcare provider practices, as well as physical environment. It is possible that health care providers in the north Texas area “see” asthma more than health care providers in other areas or are more likely to label the constellation of signs and symptoms they find as “asthma” rather than “reactive airways disease” or “bronchitis.” 

Low annual household income did not predict asthma cases in this sample. Five percent of respondents reported annual household incomes less than $20,000. This contrasts with data reported by other surveys for the US population, such as the Consumer Expenditure Survey for 2010 which found about 20% of families claiming an annual income of less than $20,000 [15]. The official United States poverty level in the year this survey was administered (2009) was $21,954 for a family of four, but many experts acknowledge that calculations of poverty guidelines in the USA are flawed resulting in substantial underestimates of current costs of supporting a family [16]. 

**Figure 2:**
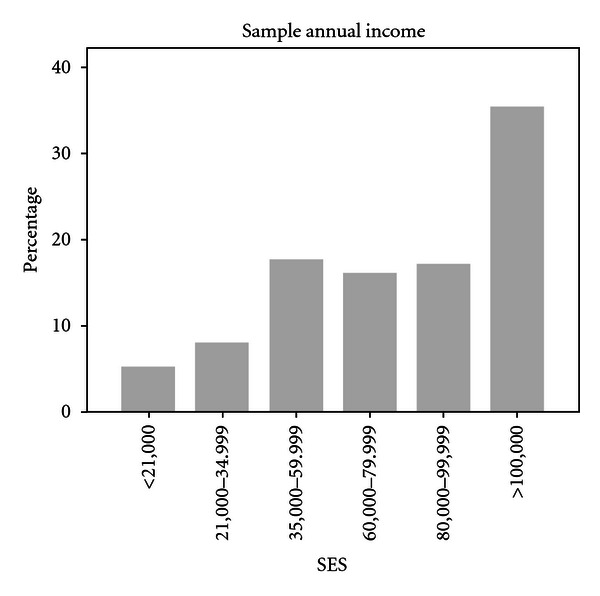
Annual income reported by respondents.

If a more realistic reference is applied, a higher annual income may reflect “low-income” better. In this survey, about 13% of respondents reported an annual household income of less than $35,000, which we believe is more likely to reflect “low-income.” This compares to 23% of USA households in metropolitan statistical areas reporting the same level of income nationally in 2009 [15]. Over half of respondents in this survey reported household incomes greater than $80,000 and a third reported incomes greater than $100,000 (Figure 2). It is possible that the north Texas area included in this survey is more affluent than other areas of the United States, which would explain the sample characteristics, but it is also possible that the survey experienced a response bias with disproportionately fewer lower-income individuals participating, thus the finding that income does not predict asthma diagnosis should be replicated by other means.

The sample suggests that the DFW Metroplex area is similar to the state in regards to children enrolled in public insurance programs. The participants in the subsample who were asked about insurance coverage (*n* = 3,271) reported 13% enrolled in Medicaid or CHIP, whereas in the year the survey was administered, the Texas Health and Human Services Commission (THHSC) reported the percentage of Texas (adults and children) enrolled in Medicaid as 13% with the great majority of recipients being children [17]. What may not be reflected well in the survey is children without any insurance coverage, which was estimated to be about 17% in 2009 [17]. 

Published estimates suggest that asthma is an expensive disease for the United States with direct and indirect costs up to approximately $20 billion [18, 19]. The finding that children with asthma in this sample visit the ER more frequently but have significantly fewer days of inpatient treatment than other children indicates that a substantial part of the direct cost for the child population with asthma is devoted to emergency room care. Children with asthma become hospitalized at times, but compared to children with other diagnoses, they spend less time in the hospital and more time in emergency rooms. Their more frequent visits for acute care may explain the small excess prevalence of health insurance, particularly Medicaid and CHIP, for children with asthma, whose parents are confronted with, and may expect, frequent medical bills for management of childhood asthma and who may be assisted to access the public insurance system by personnel in acute care settings. 

The coexistence of medical, psychological, or academic problems may have significant impact on the life of children with asthma, yet comorbidities are often unrecognized and undertreated, contributing to poor quality of life. Allergic rhinitis is the most obvious case in point. If the airway is understood as a unified entity, then events in the upper airway may be expected to influence the behavior of the lower airway [20]. Although allergic rhinitis and asthma can occur independently, they share neurologic and inflammatory pathways [21]. In this sample, the strong association between upper airway and lower airway disease was evident. Though the existence of allergic rhinitis with atopic asthma strongly influences asthma outcomes and healthcare costs, families and clinicians alike may view nasal allergies as trivial resulting in overlooking screening and treatment for this important disease [22, 23]. Primary care clinicians should include rhinitis symptoms in the health history and should include inspection of nasal passageways, turbinates, septum, and secretions in the physical examination of every patient with asthma. If chronic rhinitis is suspected, an integrated therapeutic approach as recommended by the ARIA consensus statement [21] with close monitoring of upper and lower airway symptoms and signs is indicated. 

The strong association between asthma and tonsillar hyperplasia or tonsillectomy has been noted in previous research, but few clinicians seem aware of the association and hypotheses explaining the relationship have not been well tested. Possibly tonsillar infections trigger asthma exacerbations and frequent throat infections might precede a diagnosis of asthma, but it is just as likely that tonsillar hyperplasia and asthma are linked through systemic inflammatory processes that are unrelated to throat infections [24]. A thorough assessment of the upper airway in children with asthma includes inspection of tonsil size and the history should include questions regarding snoring.

It has been suggested that up to one-third of children with asthma may have sleep-disordered breathing [25–27]. This association is also consistent with the unified airway hypothesis, which links upper and lower airway pathologies [28]. In addition, nocturnal cough is a sign of poor asthma control and may be accompanied by symptoms of poor quality sleep, such as night-time awakenings, daytime tiredness, sleepiness, and difficulties concentrating at school. There is evidence that parents perceive the quality of their school-age child's sleep as better than that described by their children, thus the results in the survey may underestimate the true degree of sleep disruption experienced by children with asthma [29]. 

The link between asthma and tonsillectomy suggests that children with asthma are more likely to have enlarged tonsils than other children and are thus more likely to experience sleep disordered breathing or obstructive sleep apnea. Clinicians who care for children with asthma routinely ask how often children's sleep is disrupted by cough, but may not be thoroughly assessing the symptoms of disordered sleep. Further evaluation, including sleep studies, should be considered for children with asthma who report poor quality sleep in spite of asthma treatment that seems to be controlling symptoms.

Strong positive associations between obesity and adult-onset asthma have been found in large, longitudinal samples, including the Nurses' Health Study and the British Cohort Study, but reports about the relationship between asthma and obesity in children have been conflicting and, like asthma, vary across geographical areas [30–34]. One problem with interpreting the extant literature is that reported associations employ different asthma indicators ranging from self-reported diagnoses to biomarkers. In this sample, children with physician diagnosed asthma were more likely to be overweight or obese than children without asthma and parents of children with asthma were more likely to perceive fewer fitness opportunities for their children than other parents. This perception may indicate anxiety-related reluctance to let children with asthma participate in the full range of available fitness opportunities. This finding also suggests that children with asthma do not exercise as much as their healthy counterparts.

Findings in this survey are consistent with the conventional belief that children with asthma use emergency rooms frequently, but episodic emergency room visits to alleviate acute symptoms is the least desirable option for management. Children with asthma need comprehensive care. A primary care home where comorbidities and associated psychosocial problems are identified and addressed is an obvious need for children with asthma that families themselves may not appreciate. Sending a consistent, regular message to families of children with asthma regarding the importance of having a medical home and visiting it regularly to monitor asthma is a feasible public health goal, while providing asthma management consistent with national guidelines is a reasonable goal for primary care practitioners. 

## 5. Conclusions

With the exception of individuals within the “plume path” of cement kilns, spatial clusters of asthma cases in this sample were not related to obvious triggers in the built environment, such as urban drilling sites, deteriorating neighborhoods, or pockets of poverty. This suggests that in this region, delivering population-based interventions and education programs targeting “inner city” and poor children may be a less efficient approach than focusing delivery of services to children with under-controlled symptoms regardless of demographic group membership. In spite of millions of dollars that have been pumped into demonstration studies of asthma education programs in schools and other community settings, controlling childhood asthma remains one of the most intransigent public health problems in the nation. Perhaps remedies as simple as increasing reimbursement for primary care visits to allow for genuine clinic-based asthma counseling and education could make an impact in Texas and elsewhere. 

Strong associations exist between asthma diagnosis and other health or social problems including allergies, tonsillectomy, sleep problems, school absences, academic problems, and behavioral problems in school. Cross-sectional data can only highlight these associations. Further investigations to unravel the causes of the identified relationships are needed. In the meantime, clinicians can use awareness of these associations to tailor management practices for children with asthma.

Limitations of the survey include possible sample bias towards relatively more affluent families and possible social desirability response bias. Because questions were split between two versions of the survey, some interesting associations, such as the relationship between asthma diagnosis and participation in the free lunch program and other social services, were not possible. In addition, response rate was low and nonresponders were not analyzed. Nevertheless, the CHHAPS survey is the largest investigation of children's health ever organized for the north Texas region and is a wealth of information that could be exploited to help guide health promotion and services delivery in the area. The survey is a model for what could be done in other regions across the country. 

## Figures and Tables

**Table 1:** Selected questions from the CCHAPS survey^1^.

Question	Possible responses
In general how would you describe this child's health?	Excellent; very good; good; fair; poor
Approximately how tall is this child?	Inches or centimeters
Approximately how much does this child currently weigh?	Pounds or kilograms
Please indicate whether a doctor or health care professional has ever told you that the child you selected (for this survey) has any of the following conditions: asthma, hay fever, skin rash or allergy, three or more ear infections, otitis media (inflammation of the middle ear), ear tubes, and tonsillectomy (had his/her tonsils removed).	Yes/no/don't know
In your opinion does the child have any behavioral, emotional, or developmental problems outside of what you would consider typical for a child his or her age?	Yes/no
Has this child ever done any of the following? Had sleep problems.	Yes/no/don't know
Please indicate how often the following items occur: people smoke cigarettes in your home.	Daily, weekly, monthly, a few times a year, seldom or never, don't know
How many times did this child visit the emergency room during the past 12 months?	———times
How many days did this child spend in the hospital in the past 12 months?	———days
What is the primary language spoken in your household?	Spanish; English; other
Has this child ever done any of the following: had academic problems at school; had behavior problems at school?	Yes/no/don't know
Approximately how many days of school did this child miss last year due to health problems?	———days

^1^Presented items are those most likely to require clarification. Wording of other items may be obtained from the authors on request.

**Table 2:** Selected family and target child characteristics.

Characteristic	Children with asthma *n* = 1350	Children without asthma *n* = 6089
Age (years) of target child	Mean 9.2 (med 9.0)	7.9 (med 8.0)
Male sex	62%	51%
African-American	9%	5%
Caucasian	69%	68%
Hispanic	17%	21%
Family annual income ≤ $35,000	13%	13%
Family received food stamps	5%	4%
Parent reports no education beyond high school	12%	15%
Parent concerned target child is overweight	15%	10%
Overweight or obese using BMI classifications	40.5%	36.5%
Academic problems at school	10%	7%
Behavior problems at school	9%	6%
Days absent from school	4.8 (med 3)	2.2 (med 1)
No ED visits in past year	68%	82%
No inpatient hospital days	92%	94%
Days spent as hospital inpatient for children who were admitted	4.31 (med 2) *n* = 102	6.38 (med 2) *n* = 384
Hay fever/allergies	79%	35%
Sleeping problems	18%	10%
Overweight/obesity	41% whole sample 41% school-aged	37% whole sample 34% school-aged
Tonsillectomy	17%	7%
No healthcare coverage	5%	7%
Medicaid or CHIP healthcare ins	16%	13%

*Percentages are rounded.

**Table 3:** Selected significant associations with asthma.

Association	OR	C.I.	*P* value
Physician diagnosed hay fever/allergies	7.2	5.9–8.8	.0001
tonsillectomy	2.9	2.3–3.8	.0001
High frequency (3+ annually) emergency room visits	2.4	1.5–3.6	.0001
Self-identification as African-American or Black	1.7	1.35–2.1	.0001
Health insurance coverage	1.5	1.1–1.8	.02

	Chi-squared		

Obesity all ages (*n* = 5864)	6.2		.01
Obesity 5 years and older (*n* = 4719)	15.2		.0001
